# Mass spectrometry imaging reveals new biological roles for choline esters and Tyrian purple precursors in muricid molluscs

**DOI:** 10.1038/srep13408

**Published:** 2015-09-01

**Authors:** David Rudd, Maurizio Ronci, Martin R. Johnston, Taryn Guinan, Nicolas H. Voelcker, Kirsten Benkendorff

**Affiliations:** 1 School of Biological Sciences, Flinders University, Bedford Park, SA 5042, Australia; 2 Mawson Institute, University of South Australia, Mawson Lakes, SA 5095, Australia; 3 Department of Medical, Oral and Biotechnological Sciences, University G. D’Annunzio, Chieti-Pescara, Italy; 4 Flinders Centre for Nanoscale Science and Technology, School of Chemical and Physical Sciences, Flinders University, Bedford Park, SA 5042, Australia; 5 Marine Ecology Research Centre, Southern Cross University, P.O. Box 157, Lismore, NSW 2480, Australia

## Abstract

Despite significant advances in chemical ecology, the biodistribution, temporal changes and ecological function of most marine secondary metabolites remain unknown. One such example is the association between choline esters and Tyrian purple precursors in muricid molluscs. Mass spectrometry imaging (MSI) on nano-structured surfaces has emerged as a sophisticated platform for spatial analysis of low molecular mass metabolites in heterogeneous tissues, ideal for low abundant secondary metabolites. Here we applied desorption-ionisation on porous silicon (DIOS) to examine *in situ* changes in biodistribution over the reproductive cycle. DIOS-MSI showed muscle-relaxing choline ester murexine to co-localise with tyrindoxyl sulfate in the biosynthetic hypobranchial glands. But during egg-laying, murexine was transferred to the capsule gland, and then to the egg capsules, where chemical ripening resulted in Tyrian purple formation. Murexine was found to tranquilise the larvae and may relax the reproductive tract. This study shows that DIOS-MSI is a powerful tool that can provide new insights into marine chemo-ecology.

Secondary metabolites are known to chemically mediate intra- and interspecies interactions between organisms^1^. In molluscs, secondary metabolites have been detected and identified during mate attraction^2^, defence^3^,^4^, predatory behaviour^5^, anti-fouling^6^,^7^ and reproduction^8^. The importance of understanding the mechanisms behind these chemical interactions within a species cannot be underestimated, particularly when specific secondary metabolites impart a competitive advantage. Advantageous secondary metabolites have been known to have community wide effects across multiple trophic levels, termed “keystone” molecules^9^,^10^. The initial step in understanding the function, and therefore relevance of secondary metabolites to the producing organism, is to understand the *in situ* synthesis, storage and deployment^11^,^12^. To place secondary metabolites in an ecological context, their biodistribution and abundance should be examined on a temporal scale relevant to the biological phenomena which they mediate. Reproducible methodologies that can spatially and temporally detect secondary metabolites *in situ* will significantly advance the field of chemical ecology^13^.

Current advances in mass spectrometry imaging (MSI) provide a sophisticated platform to spatially map the distribution of biologically-relevant molecules or mixtures by detection of their molecular mass and characteristic fragment ions in tissues^11^,^14^. MSI is often based on matrix-assisted laser desorption ionisation (MALDI)^15^,^16^, a soft ionisation technique that relies on aromatic molecules (the matrix) for energy transfer from a UV laser to the analyte. MSI has been gaining traction in the analysis of ecologically relevant chemical exchanges, demonstrating bacterial metabolic communication^17^,^18^, symbiotic interactions^11^,^19^ and chemical defense^20^. However, the standard matrix used in MALDI can suppress signals from small molecules, particularly when present in low abundance^21^. Consequently, matrix-free MSI approaches, such as desorption-ionisation on silicon mass spectrometry (DIOS-MS), which uses a thin porous silicon (pSi) film^22^, have been developed to enhance detection of small molecular weight organic compounds (typically <1000 Da), without the need for a matrix^23^,^24^. Recently, we reported a new method to detect brominated indoles from mollusc tissue using DIOS-MSI^25^. This approach uses tissue stamping onto a pSi film to extract low molecular weight molecules by affinity into the porous surface, whilst retaining the spatial distribution of secondary metabolites that occurs *in situ*. Imprinting from frozen cryo-sections also allows the spatial organisation of the tissue to be maintained, an advantage for the analysis of exceptionally soft marine secretory tissue. Exploiting DIOS-MSI to detect secondary metabolites comes with a major benefit, namely the possibility of chemically functionalising the pSi to target specific compounds or classes of compounds that would otherwise be difficult to detect^25^. Functionalised pSi therefore provides an optimal substrate for monitoring micro-changes in complex heterogeneous marine tissue, in space and time.

Muricidae molluscs have been of significant interest as biological and chemical resources since antiquity^26^,^27^. They are a source of biologically active brominated indoles (Fig. 1) that are precursors to the historically significant dye Tyrian purple^28^. Tyrian purple (6,6′-dibromoindigo) was the first marine natural product to be structurally elucidated (Friedlander 1909)^29^ and is still commonly used as a tool for teaching organic chemistry^30^. Nevertheless, the biological function of Tyrian purple remains unknown and is suggested to simply be an artefact^31^ formed from the degradation of indoxyl sulfate precursors, which are stored as salts of choline ester derivatives (e.g. murexine, Fig. 1) in the hypobranchial gland of these molluscs^32^. Tyrian purple production is initiated by reaction of the indoxyl sulfate precursors with an aryl sulfatase enzyme (Fig. 1), which is also produced and stored in the mollusc^33^, thus suggesting a regulated ecological function for the precursors. Muricidae choline esters show marked neuromuscular blocking activity^34^ and have been implicated in the paralysis of prey by these predatory molluscs^34^, whereas the brominated indole intermediates have antibacterial activity and have been implicated in the defense of the egg capsules^35^. The brominated indoles have also been found in extracts of the reproductive organs^36^, suggesting a maternal source for the egg capsules. However, it is unclear if the choline esters are also transferred into the egg capsules or why the molluscs constitutively produce and store these two distinct classes of compounds as an indoxyl sulfate-choline ester salt in the hypobranchial glands for controlled release on reaction with aryl sulfatase.

Here, we report the *in situ* spatial identification of brominated indoles and choline esters and examine temporal changes in their distributions within adult *Dicathais orbita* (Muricidae, Neogastropoda, Mollusca). MSI analysis was applied at different stages of the reproductive cycle of mature female *D. orbita,* along with early and late stages of encapsulated larval development, to investigate the role of these two classes of secondary metabolites in reproduction. Based on these results we hypothesise new biological roles for murexine in the reproduction and larval development of Muricidae. The tranquilising effect of murexine on the encapsulated larvae was then confirmed in biological assays. Overall, this approach allows spatial and temporal changes in secondary metabolites to be defined within the context of biological processes, such as reproductive activities.

## Results and Discussion

### Detection and identification of secondary metabolite patterns on functionalised pSi

**Figure 2:**
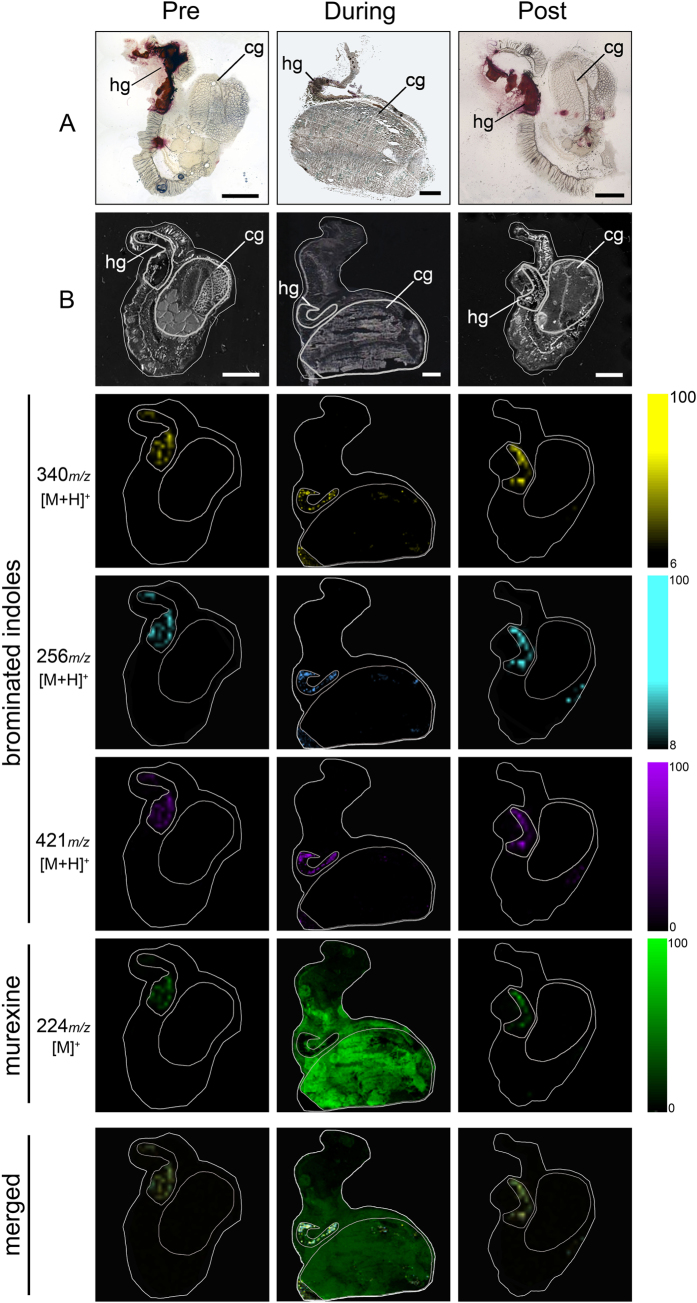
DIOS-MSI maps of secondary metabolites imprinted onto pSi from female *D. orbita* across the reproductive cycle, in positive ion mode at 100 μm spatial resolution. (Pre) representative female section sampled 30 days prior to the breeding season. (During) female section sampled during encapsulation. (Post) representative female section sampled 14 days post encapsulation. Maps are compared to (**A**) histological sections and (**B**) scanned tissue sections on pSi prior to removal. Tissue regions include (hg) medial hypobranchial gland and (cg) capsule gland. Ion maps *m/z* 340 corresponds to tyrindoxyl hydrogen sulfate [M+H]^+^, *m/z* 256 to tyrindoleninone [M+H]^+^, *m/z* 421 to Tyrian purple [M+H]^+^, and *m/z* 224 to murexine [M]^+^. Scale bar set to 2 mm.

**Figure 3:**
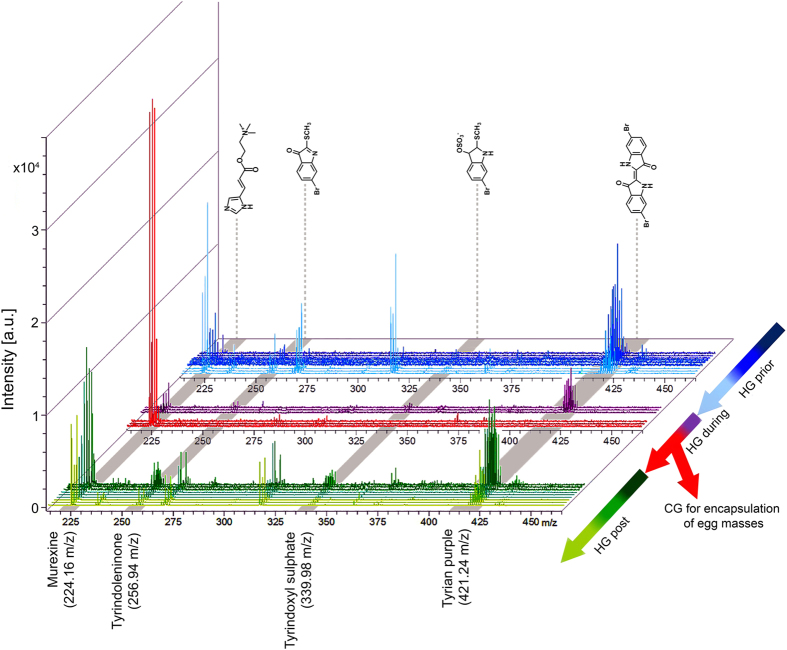
*D. orbita* secondary metabolite mass spectra from across the reproductive cycle. Spectra show mono- and dibrominated ion clusters for the brominated indoles and murexine detected using DIOS-MSI in positive mode from the medial hypobranchial gland and capsule gland from reproductively active females. (HG) hypobranchial gland; (CG) capsule gland.

To explore the biological roles of muricid choline esters and brominted indoles using DIOS-MSI in the context of egg laying and maternal investment, established spatial patterns based on previous histochemical staining^37^ were used as a basis to track changes in secondary metabolite distribution at different phases of the reproductive cycle. The reliable performance of pSi in detecting the range of secondary metabolites was validated using organic extracts prepared from the same population of breeding individuals. Extracts were analysed by liquid chromatography mass spectrometry (LC-MS/MS) according to established procedures in our laboratory^35^,^36^,^38^ (Supplementary Figs 1 & 2), and used as a comparison for those detected in DIOS-MS and MSI (Figs 2 and 3). The capacity for pSi to desorb and ionise brominated indoles from the tissue samples was confirmed by the detection of isotopic patterns in MS/MS analysis consistent with the crude extracts analysed by LC-MS (Supplementary Figs 2 and 3) and with purified compounds and synthetic 6 bromoisatin and 6,6′-dibromoindigo spotted directly onto the pSi surface^39^. DIOS-MS takes advantage of the van der Waals interactions and hydrogen bonding between the analyte containing the secondary metabolites and the silanised pSi surface. Attractive properties combined with high porosity (~600 m^2^ cm^−3^) allow pSi to selectively extract small molecules in a sponge-like manner^40^. Consistent with our previous studies^25^,^39^, brominated indoles across a broad range of polarities in the low mass region from *m/z* 256–421 (Fig. 2 and Supplementary Fig. 2), showed affinity to the pSi surface.

Stamping allowed low mass metabolite transfer from cryostat sections of the tissue to the pSi (Fig. 2), whilst the hydrophobic pSi surface facilitated the subsequent removal of residual tissue by pipetting with water, with minimal delocalization of the secondary metabolites. The removal of larger biological material reduces spectral complexity by eliminating fragments from highly abundant larger compounds like membrane lipids, which can suppress low abundant target signals. The isotopic clusters for mono- and dibrominated indole structures allow for the assignment of brominated secondary metabolites detected in DIOS-MS, including the hydrophilic dye precursor tyrindoxyl hydrogen sulfate (duplet ion cluster at *m/z* 339, 341 [M+H]^+^), the intermediate tyrindoleninone (duplet ion cluster at *m/z* 256, 258 [M+H]^+^), and the least soluble end product Tyrian purple (triplet ion cluster centered at *m/z* 421 [M+H]^+^) (Fig. 3, Supplementary Fig. 2).

DIOS-MS was also effective in detecting the choline ester murexine (urocanylcholine; major ion *m/z* 224 [M]^+^, Fig. 3, Supplementary Fig. 3), in the first spatial analysis of this well studied^34^ secondary metabolite. The presence of murexine was confirmed by extraction from the remaining tissue of a reproductively active female and was structurally elucidated using LC-MS/MS, ^1^H-NMR and ^13^C-NMR fingerprinting (summarised in Supplementary Table 1), and confirmed by comparison to choline and choline ester salts of tyrindoxyl sulfate previously reported from *D. orbita* and other muricidae molluscs^32^,^41^. Apart from major ion *m/z* 224, murexine showed major fragment ions in LC-MS/MS at *m/z* 165 and 121 (Supplementary Fig. 3). These same fragment ions were detectable in DIOS-MS spectra co-localised with the major ion *m/z* 224 (165 and 121; Supplementary Fig. 4), confirming the detection of murexine using post source decay, and more broadly demonstrating the accuracy of DIOS-MS in the low mass range, whilst eliminating matrix suppression and non-target spectral “noise”.

### Biodistribution of secondary metabolites across the reproductive cycle

A major change in the biodistribution of Muricidae secondary metabolites is clearly apparent across the reproductive cycle of female *D. orbita*, with murexine, in particular, moving from the hypobranchial glands to the capsule glands during the breeding season (Figs 2 and 3). Furthermore, there is a substantial increase in the intensity of murexine detected in egg laying females, in comparison to the pre- and post- breeding stages (Figs 2 and 3). This suggests that the choline ester murexine may play a fundamental role in the reproduction of Muricidae molluscs. A role in reproduction has been previously suggested for the brominated indole precursors of Tyrian purple^31^,^36^, whereas the choline esters have been suggested to play a role in the feeding activities of these predatory snails^31^,^34^ and/or simply assumed to stabilise the indoxyl sulfate precursors as a salt for storage within the hypobranchial gland tissue^28^,^32^. Here DIOS-MSI has provided novel information that suggests an up-regulation of choline esters for reproduction, that may in fact be far more important for Neogastropods than previously anticipated.

**Figure 4:**
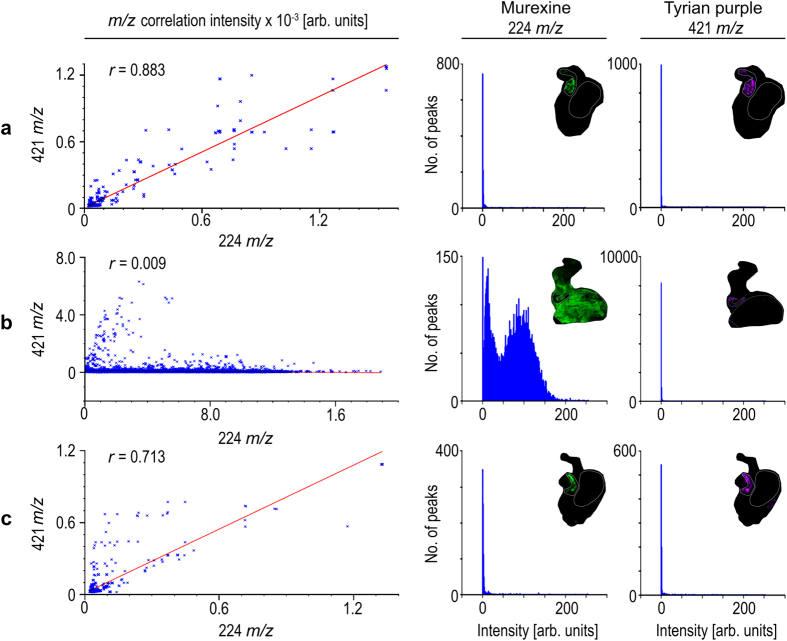
Representative correlation plots (left hand panels) for co-localised *m/z* patterns for murexine against Tyrian purple with corresponding intensity histograms of murexine (middle panels) and Tyrian purple (right hand panels) spot spectra generated in SCiLS Lab imaging software (Bremen, Germany). Where Tyrian purple and murexine co-localise within; (**a**) pre-reproductive female tissue section; (**b**) reproductive female tissue section; and (**c**) post reproductive female tissue section, with intensities for murexine and Tyrian purple.

The co-localisation of murexine with the brominated indoles, tyrindoxyl sulfate and Tyrian purple in the medial region of hypobranchial glands (Fig. 3) was confirmed by correlation analysis of DIOS-MSI for the pre-reproductive females (R-squared 0.883 ± 0.1; Fig. 4a) and post-reproductive females (R-squared 0.713 ± 0.27; Fig. 4c). However, during the spawning (egg deposition) stage of the reproductive cycle, there was a substantial increase in the intensity (Fig. 3) and spatial detection of murexine in the capsule gland (Fig. 2), but not in the brominated indoles. Consequently, there was no longer a correlation between these secondary metabolites in egg laying females (R-squared 0.009; Fig. 4b). The increase in murexine in the capsule gland during egg deposition appears to be at the expense of the medial hypobranchial gland (Fig. 2 and Supplementary Fig. 5). This implies that availability of murexine for feeding activities^31^,^34^ is reduced during spawning. Few studies have investigated the energetic cost of maternal provisioning for non-primary metabolites^1^, and MSI may provide an attractive method for tracking mother to offspring transfer.

### Maternal provisioning and chemical changes during encapsulated larval development

**Figure 5:**
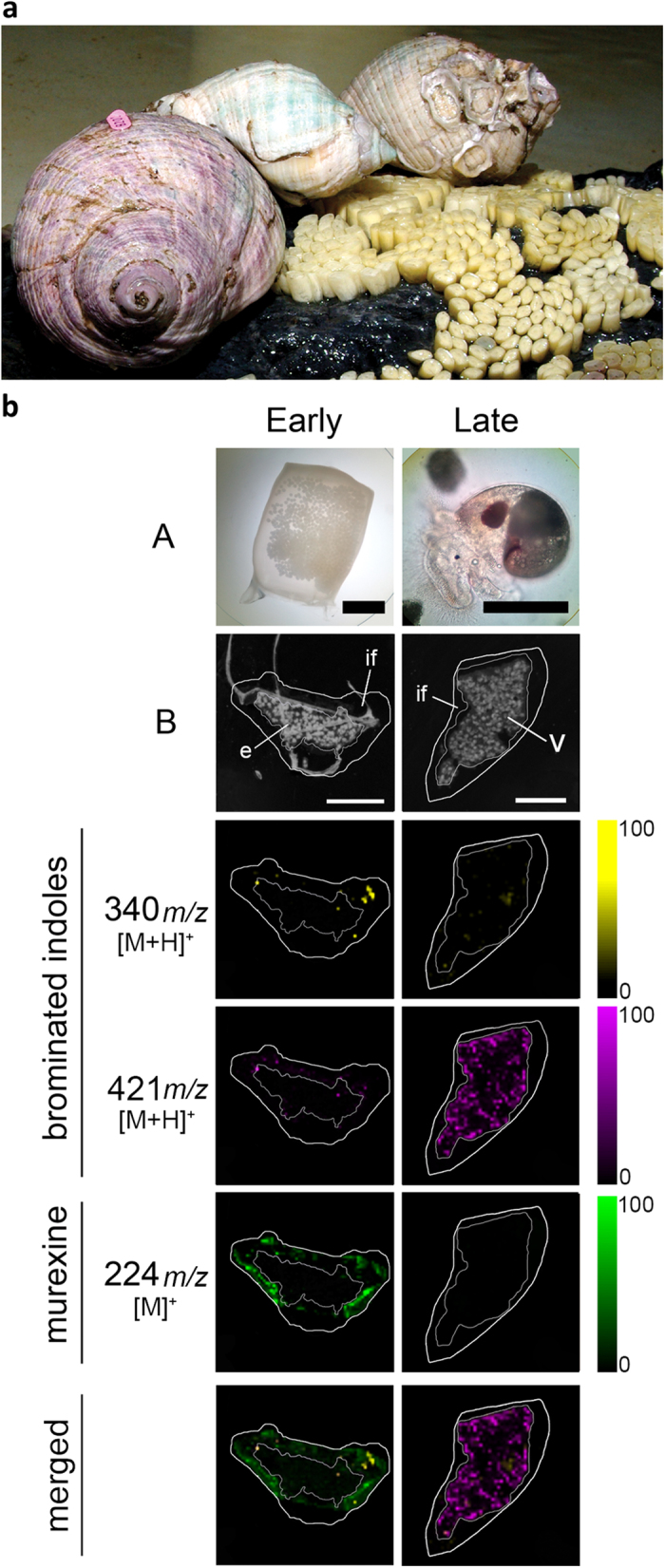
*D. orbita* during egg deposition and DIOS-MSI maps of egg capsules across the developmental period, in positive ion mode at 100 μm spatial resolution. (**a**) Reproductive adults during the encapsulation of larvae and early stage capsules adhered to substrate (photo by Rudd, D.). (**b**) early stage capsule sampled immediately post deposition (left panels A = whole capsule) and late stage capsule after 35 days post deposition (right panels A = encapsulated veliger larvae). DIOS-MSI of the secondary metabolites are compared to (**B**) scanned cross sections of the egg capsules stamped onto pSi prior to removal. Labels on the imaged regions include; embryo mass (**e**), intracapsular fluid, (if) and veligar (v) stage larval mass. Ion maps *m/z* 340 corresponds to tyrindoxyl hydrogen sulfate [M+H]^+^, *m/z* 421 to Tyrian purple [M+H]^+^, and *m/z* 224 to murexine [M]^+^. Scale bar set to 1 mm.

DIOS-MSI on the egg capsules (Fig. 5) confirmed that murexine is transferred from the female capsule gland into the egg capsules of *D. orbita*, which is the first report of murexine in Neogastropod egg capsules. DIOS-MSI detected murexine only in the intracapsular fluid surrounding the early stage embryos in freshly laid egg capsules, where it is co-localised with tyrindoxyl sulfate and some minor intermediate brominated indoles and end products (Fig. 5b). In the capsules with late stage veliger larvae, water soluble murexine was no longer detectable. Tyrindoxyl sulfate also decreased in intensity and distribution during encapsulated larval development (Fig. 5, Supplementary Fig. 7). Conversely, insoluble Tyrian purple increased in the late stage capsules but was barely present at the early stage. This provides evidence for a unique form of chemical ripening in the egg capsules^19^ involving two distinct classes of secondary metabolites specific to the Muricidae family of marine molluscs. The apparent high abundance of murexine relative to tyrindoxyl sulfate in breeding snails implies a naturally selected role for this compound during reproduction and early stage development, whereas tyrindoxyl sulfate could be acting as a controlled release counter ion to murexine within the egg capsules and hypobranchial gland. In this case, the formation of antimicrobial brominated indole intermediates during intracapsular development^35^ could be a secondary function fortuitously acquired over the course of Muricidae evolution.

### Biological role for murexine

The purpose of murexine within the female capsule gland is open to interpretation. However, since this compound is a potent muscle relaxant^34^, it may help relax the reproductive tract during egg deposition. Murexine has been suggested as a ligand for the muscle type nicotinic-acetylcholine receptor^34^ (nAChR) based on the pharmacological actions of murexine *in vivo* within vertebrates^34^,^42^,^43^. The *in vivo* paralytic effect of murexine has the greatest similarity to suxamethonium, a muscle type nAChR agonist^34^. The choline esters are expected to become biologically active only when not complexed with tyrindoxyl sulfate^41^, as generally the quaternary ammonium ion of choline esters contribute to receptor binding and are required for the tranquilising activity for a range of other choline ester structures and acetylcholine^44^,^45^. The detection of murexine in the absence of brominated indoles indicates active muscle relaxing effects in the capsule gland environment.

**Figure 6:**
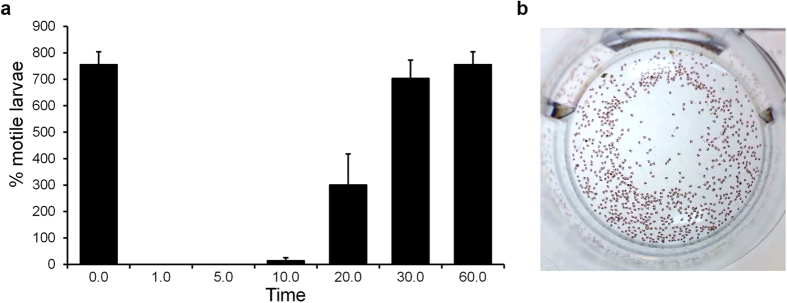
The proposed biological role of murexine in the *D. orbita* egg capsules using a larval motility assay in the presence of 50 ppm murexine extract: (**a**) percentage of motile larvae counted using short 30 s videos over 60 min and (**b**) a video still shot of larvae at time 0 prior to the addition of murexine extract (video online: https://youtu.be/rlCvyyhnXAE).

The role of murexine within the egg capsules as a natural tranquiliser during larval development is also plausible. To test this, we purified murexine at 0.22 mM (50 mg/L) and confirmed that it has a temporary tranquilising effect on late stage intracapsular larvae lasting over 60 minutes (Fig. 6a and Supplementary Video 1). Developing larvae shells are delicate and oxygen is limited during muricid intracapsular development^46^, despite this period being a metabolically active period. *D. orbita* reproductive females could be provisioning offspring with intracapsular tranquilisers to ensure they develop ready for life in the open sea. Indeed nearly 100% hatching success rate has been reported for the thousands of delicate larvae contained within each egg capsule of *D. orbita*^47^. Natural tranquilisers have been previously reported in molluscan egg masses, specifically in the common squid *Loligo vulgaris,* which incorporates an unidentified natural tranquiliser in perivitalline fluid within egg cases^48^, preventing premature hatching. Although the bioactive compound in squid perivitalline fluid does not appear to have been identified to date, cephalopods do not possess a hypobranchial gland and there are no reports of choline esters in the venom glands, ink or chemical messengers involved in the reproduction of cephalopods, despite extensive studies^34^,^49^,^50^. Therefore, the new biological role proposed here for murexine, appears to be an interesting example of functional convergent evolution between the gastropods and cephalopods. These two classes of marine molluscs have independantly evolved the deposition of benthic masses, as well as chemical sedatives to protect the encapsulated larvae.

Overall, this study has confirmed the usefulness of MSI for providing new insights into marine chemical ecology. DIOS –MSI has been used to map the distribution of two distinct classes of compounds over the adult reproductive and encapsulated larval stage in a muricid mollusc, revealing a significant upregulation of muscle relaxing choline esters in the capsule gland of females during egg deposition, and defining a role for this compound in the egg masses during the earlier stages of intracapsular development. The co-localisation of murexine with tyrindoxyl sulfate in both the hypobranchial glands of adults and in the early stage egg capsules confirms the intrinsic link between these secondary metabolites and provides evidence for a novel chemical ripening system that is likely to play a fundamental role in the encapsulated development of Muricidae larvae.

## Methods

### Collection and maintenance of whelk breeding population

Pursuant to section 115, *D. orbita* samples were collected using an Exemption Permit to the South Australian Fisheries Management Act 2007 section 70 under the exemption number 9902638.

Prior to the start of the breeding season, adult *D. orbita* were collected from rocky intertidal shores on Southern metropolitan coast in South Australia and housed in recirculating aquarium systems at Flinders University. The breeding population was maintained at temperate marine conditions (18 °C and 35 psu seawater) and fed *ad libitum* on a diet of bivalves. Conditioned boulders were provided for egg capsule deposition. Pre-reproductive adult females (*n* = 3) were selected for mass spectrometry imaging (MSI) to analyze secondary metabolite distribution 30 days prior to the standard breeding time (early September). During the breeding period, a female (*n* = 1), observed in the process of egg capsule deposition was selected for MSI. Post-reproductive females (*n* = 3), two weeks after egg capsule deposition, were selected to image the post-reproductive tissue. Duplicate (*n* = 2) egg capsules deposited from reproductive females were selected immediately after deposition to assess early stage embryos and maternal capsule contents by MSI. The remaining capsules were maintained for full intra-capsular development of 35 days, after which two (*n* = 2) capsules with actively swimming larvae were selected to assess the veliger larvae and late stage capsule contents by MSI.

### Tissue preparation for mass spectrometry imaging (MSI)

Selected adult specimens were prepared by cracking open the shell with a vice at the junction between the primary body whorl and spire. The soft body was then removed by cutting the columnar muscle. Soft tissue was rinsed in MilliQ water to reduce residual salt. Female hypobranchial glands and pallial gonoduct, including the egg capsule glands, were removed by incision along the connective mantle tissue between the ctenidium and the branchial hypobranchial gland, along the posterior gonoduct and digestive gland. The hypobranchial gland and pallial gonoduct were left connected and were placed in 5 mL polypropylene cryo-vials (Sarstedt, Nümbrecht, Germany) and snap frozen in liquid nitrogen. Frozen tissue samples were protected from light and stored at −80 °C until required.

Egg capsules were retrieved from the substrate by an incision underneath the basal membrane of the capsule wall, to maintain capsule integrity, rinsed in MilliQ filtered water and snap frozen in liquid nitrogen within 5 mL cryo-vials for storage at −80 °C until required.

### MSI pSi substrate fabrication, oxidation and functionalisation

Monocrystalline (0.008–0.02 Ωcm) antimony doped n-type Si (100) wafers (Silicon Quest International, CA, USA) were cut, methanol sonicated for cleaning and dried prior to substrate fabrication by light-assisted anodic etching^23^. Photopatterned pSi arrays were secured in a custom built Teflon cell in contact with a gold foil anode (Space Products International, CA, USA), with platinum wire (0.5 mm, 99.9%; Aldrich, WA, USA) shaped into a ring acting as a cathode. The teflon cell was then filled with an electrolyte solution of 1:1 hydrofluoric acid (HF): ethanol. The submerged Si surfaces were illuminated using a fiber optic light source passing through a set of two aspheric lenses, f = 80 mm (OptoSigma, CA, USA) for collimation. A 20 mA constant current was then applied across the cell for 2 min via a source meter program, constructed in LabView 6.1 to operate a 2425 current source meter (Keithley, Ohio, USA). Fabricated pSi were washed several times with methanol prior to being dried under nitrogen gas.

Freshly etched pSi were ozone-oxidised with a flow rate of 3.25 g/hr using an Ozone-Generator 500 (Fischer, Germany). After oxidation, pSi were subjected to a second pore broadening etch with 5% HF/H_2_O for 30 s. The double etched pSi surfaces were ozone oxidised as above. Etched hydroxy-terminated pSi surfaces were then silanized using 80 μL of neat silane (F5PhPr) for 15 min at 90 °C. Silanized pSi arrays were washed with methanol, dried under nitrogen gas and stored in a dessicator until required.

### Tissue sectioning and imprinting for DIOS-MS

Hypobranchial glands (with connected pallial gonoduct) were mounted on cryo-section specimen holders and fixed into place with a minimal amount of embedding medium (Optimum Cutting Temperature Compounds (OCT); Tissue-Tek), on the base away from the target tissue. The hypobranchial gland with connected pallial gonoduct was serially transverse cryo-sectioned (Leica 1800 Cryostat, Leica Microsystems) until the mid-region of the medial hypobranchial gland was exposed. Sections to be imaged contained both medial hypobranchial tissue and attached capsule gland tissue. A 15 μm thick cryo-section was placed on a glass slide for optical imaging using light microscopy (Zeiss Axio Imager Compound Microscope and Axio Imaging software). The serial 15 μm thick cryo-section was placed on a functionalised pSi chip for tissue imprinting and kept for 30 minutes at room temperature in a desiccator to promote tissue analyte-surface interaction and extract small molecules by affinity. Imprinted pSi chips were digitally scanned using a conventional desktop scanner (Epson V700 Photo Scanner). Prior to MSI the residual tissue on the pSi surface was removed by immersion in MilliQ at 70 °C for 10 minutes. Tissue removal was aided with a gentle stream of hot water from a pipette, dried and rinsed twice in fresh MilliQ water.

Egg capsules were mounted on cryo-section specimen holders on an anterior-posterior axis and fixed in place with a minimal amount of OCT. Capsules were serially sectioned down to half width, removed from the specimen holder and stamped onto pSi chips for 30 minutes in a desiccator for capsule analyte-surface interaction at room temperature. Imprinted pSi chips were digitally scanned (Epson V700 Photoscanner) and remaining tissue was removed as above.

### DIOS-MS and MSI

Imprinted pSi chips were mounted onto a customised MTP 384 ground steel target plate (Bruker-Daltronics GmbH, Bremen, Germany), secured with conductive carbon tape, and loaded into an Autoflex III TOF/TOF mass spectrometer (Bruker-Daltronics) equipped with a SmartBeam 200 Hz laser. Scanned tissue images, on pSi substrate prior to tissue removal, were loaded into FlexImaging 2.1 (build 25) and aligned with the steel target plate containing the pSi sample based on three teaching points. A small margin around each tissue area was allowed for capillary movement of fluids out of the tissue. FlexImaging 2.1 distribution maps were used to control FlexControl 3.3 (build 85) during image acquisition.

Quadratic external calibration of the TOF tube was performed prior to each acquisition using α-cyano-4-hydroxycinnamic acid (CHCA) adducts together with bradykinin (1–7) and angiotensin II, spotted on a free area of the pSi substrate. Specifically, monoisotopic peaks for the calibration range included: K^+^ 38.9637, CHCA [M+H-H2O]+172.0399, CHCA [M+H]+190.0504, CHCA [M+Na]+212.0324, CHCA [2M+H-CO2]+335.1032, CHCA [2M+H]+379.0930, Bradykinin Fragment 1–7 [M+H]+757.3991 and Angiotensin II [M+H]+1046.5418.

Samples were run in reflectron positive mode in the 20–1000 Da range, with a spatial resolution of 100 μm and medium laser focus, corresponding to a 50 μm diameter. Mass spectrometer settings: Ion source 1–19.00 kV, ion source 2–16.80 kV, lens−8.25 kV, reflector 1–21.00 kV, reflector 2–9.40 kV. Data sets were further processed by baseline subtraction (1) and normalised threshold settings (0.5).

Ion peak distribution maps were generated in FlexImaging 2.1 and mass spectra were analysed in FlexAnalysis 3.3. Resulting masses associated with known compounds were assigned colours for visual localisation with reference to histological sections.

To calculate co-localisation of mass spectra across all tissue samples from DIOS-MS imaging, data files were imported into SCiLS Lab (Bremen, Germany) and run through an unsupervised cluster analysis of spatially similar *m/z* distribution patterns providing a summed spectra of peaks that co-localise within tissue regions. Correlation analysis identified where brominated indole distribution patterns overlapped with murexine based on the reproductive cycle. The peak distribution correlation of representative samples were plotted with corresponding peak intensity for each *m/z* value.

### Extraction and elucidation of brominated indoles

Fresh hypobranchial glands (6.58 g) were solvent extracted^35^. Glandular tissue was dissolved in an equal portion of chloroform and methanol (1:1 v/v, Sigma, CHROMASOLV®, HPLC grade) and continuously stirred overnight. After vacuum filtering (Whatman filter paper 1), the polar and lipophilic fractions were separated using 20 mL milliQ water. The chloroform fraction contained the intermediate precursor brominated indoles, whilst the methanol/water fraction contained the ultimate precursor to Tyrian purple, tyrindoxyl sulfate and tyrindoleninone. Each extract fraction was evaporated to dryness under reduced pressure of 470 mbar at 40 °C on a Rotavapor® R-114 (BÜCHI Labortechnik AG, Flawil, Switzerland), weighed (214.7 mg) and then re-dissolved in 1 mL of acetonitrile (Sigma, CHROMASOLV®, HPLC grade) within amber vials for LC-MS analysis.

Brominated indoles were separated with a Waters 2695 high performance liquid chromatographer (HPLC; Waters Alliance®) coupled to a mass spectrometer (MS; Micromass Quattro micro™ tandem quadrupole MS System, Waters, Milford, MA, USA) for identification. HPLC separation was performed on a reverse-phase hydrophobic column (Synergi™, Hydro-RP, 4 μm C_18_ phase, 80 Å, 250 × 4.6 mm i.d., Phenomenex, Lane Cove, NSW, Australia) according to the previously reported elution gradient^36^ of acetonitrile in water with 1% formic acid at a flow rate of 300 μL/min with parallel UV/Vis photo-diode array (PDA) detection at 300 and 600 nm. Electrospray ionisation (ESI-MS) facilitated the identification of brominated indole structures and data were analysed using the Masslynx 4.1 data system (Waters). Retention times were standardised using 40 μM synthetic 5-bromoisatin (Sigma-Aldrich, technical grade) in acetonitrile, a structural isomer of the Tyrian purple precursor 6-bromoisatin. The identification of brominated indoles was based on peak retention time, expected mass and isotopic clusters for the mono- and dibrominated compounds within mass spectra, with reference to previously published structures for this species^26^,^32^,^35^,^51^, listed in Table S1.

### Extraction and elucidation of murexine structure

The remaining frozen tissue (1.3 g) from the reproductive female, after MSI tissue collection, was used for extraction of reproductive female-derived murexine to ensure specificity of detection in DIOS-MSI. Murexine was extracted three times in 30 mL volumes of acetone and pooled. The extract was vacuum filtered through a PTFE membrane filter (pore size 0.2 μm), evaporated to dryness, and washed three times with 10 mL of diethyl ether (Ajax Finechem, AR grade) to remove fats^18^. Total extract was then taken up in 5 mL of acetonitrile (ACN) for thin layer chromatography (TLC) detection^18^, LC-MS and NMR analysis.

TLC was used for the initial detection of murexine, based on previous reports of choline ester compounds in members of the Muricidae^34^. Approximately 5 μL of extract was collected into glass capillary tubes (32 mm, 0.6 mm i.d., BLU-TIP®), spotted on aluminum-backed silica TLC plates (F60, Merck) and separated using an n-butanol-ethanol-acetic acid-water (8:2:1:3) gradient for the mobile phase. Visualisation of the choline ester spots was achieved using Dragondorff reagent (Fluka, Sigma-Aldrich Chemie GmbH) according to the retention times previously reported^34^.

Three replicate 10 μL subsamples of the capsule gland extract were subjected to ultra-performance liquid chromatography (UPLC)-MS, based on a modified LC-MS protocol for detection of acetylcholine^52^. Separation was provided by an Acquity UPLC system (Waters), 10 μL injection volume, on a reverse-phase column (Atlantis T3, 3 μm C_18_, 3 × 100 mm i.d., Waters) using 0.5% formic acid (A) and acetonitrile (B) at a flow rate of 0.5 mL/min (gradient of solvent: 0–10 min, 98% A and 2% B), with UV PDA. ESI-MS detected the murexine structure on the Micromass Quatro micro™ tandem quadrupole mass spectrometer and MS and UV data was acquired using Masslynx 4.1 data system (Waters). To see the structural features of murexine, in ESI, a scan at 20 V was compared to a collision induced dissociation (CID) scan at 35 V cone voltage (positive ion electrospray, 80 to 500 *m/z* mass range).

The remaining reproductive female tissue extract was used for ^1^H-NMR and ^13^C-NMR fingerprinting for identification of reproductive female capsule gland extract components. The bulk of contaminants were removed by solid phase extraction column (Prevail™C_18_ reversed-phase, 4 mL 500 mg packed bed, Grace, Deerfield, IL, USA), resuspended in methanol-d_4_ followed by ACN-d_3_ in 5 mm (600 MHz) NMR tubes (Bruker-Daltronics). Chemical shifts were recorded on a 600 MHz NMR spectrometer (Bruker Avance, Karlsruhe, Germany) using an inverse multinuclear probe (5 mm) and a triple resonance HCN probe and referenced to residual solvent peaks. Structural confirmation of individual compounds was elucidated using ^1^H-NMR and ^13^C-NMR chemical shifts and correlation analysis. Chemical shift assignment of murexine was based on similarity to previously reported ^1^H-NMR from murexine (in a mixture of 90% ACN-d_3_)^53^ and ^1^H-NMR for tyrindoxyl sulfate and the complex in methanol-d^32^. Reproductive derived murexine was validated against a semi-purified acetone extract from adult hypobranchial gland extractions (106 mg).

### Bioassay of murexine in hatching stage *D. orbita* larvae

To assess the effect of murexine on larval motility, fresh hypobranchial glands were extracted (127 g) using acetone. Fresh glands were extracted overnight, with continuous stirring, in 200 mL of acetone and remaining tissue further extracted in 200 mL overnight. The supernatant was collected, pooled, then vacuum filtered through a PTFE membrane filter (0.2 μm) and evaporated to dryness. The extract was resuspended in 30 mL of MilliQ filtered water. Fats were removed by three washes with an equal portion of diethyl ether. Murexine was separated from the bulk of contaminants using a solid phase extraction column (Prevail™C18 reversed-phase, 4 mL 500 mg packed bed, Grace, Deerfield, IL, USA) and a purified fraction of the choline ester was collected by concentration on a normal-phase SPE column (Alltech® normal-phase silica column, 4 mL 500 mg packed bed) eluted with successive additions of 10, 30, 40, 50, 60, 70, 80, 95, 99% ethanol. Fractions of 10–30 mL were collected and run on TLC for purity. The fractions containing murexine were evaporated to dryness and resuspended in artificial seawater to collect murexine for bioassays.

Late stage intracapsular *D. orbita* larvae were subjected to semi-purified murexine at a concentration of 50 ppm and motility was recorded over a 60 min motility assay. Replicate (*n* = 12) assays were run in 24 well cell culture plates using 4 mL of artificial seawater. Seawater was pipetted onto larvae just prior to the addition of murexine to ensure larvae were actively swimming. Murexine in artificial seawater (0.25 mg in 1 mL) was added slowly to larvae (in two amounts) and swimming activity was assessed by short 30 s video recordings at timed intervals 0, 5, 10, 20, 30 and 60 min. Movement by beating cilia across still shots during short videos were scored as swimming.

## Additional Information

**How to cite this article**: Rudd, D. *et al.* Mass spectrometry imaging reveals new biological roles for choline esters and Tyrian purple precursors in muricid molluscs. *Sci. Rep.* **5**, 13408; doi: 10.1038/srep13408 (2015).

## Supplementary Material

Supplementary Information

Supplementary video

## Figures and Tables

**Figure 1:**
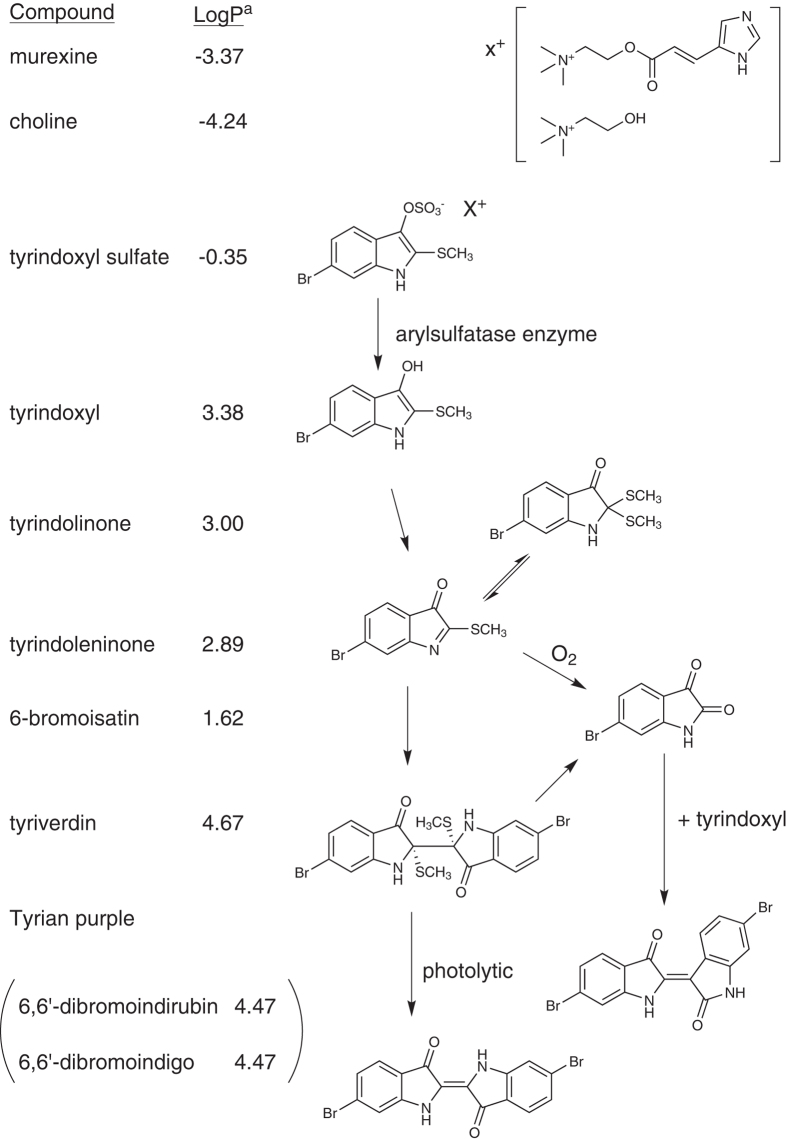
The enzymatic, oxidative and photolytic reaction of bioactive compounds found in *D. orbita* hypobranchial glands (Muricidae, Mollusca) with corresponding solubility indicator. aLog P was calculated using the chemoinformatics software Molinspiration.
