# Supplementary material for: Mass spectrometry imaging reveals new biological roles for choline esters and Tyrian purple precursors in muricid molluscs
Source: Sci Rep. 2015 Sep 1;5:13408. doi: 10.1038/srep13408 (PMC4555103; doi:10.1038/srep13408)
Supplement: Supplementary Information [file srep13408-s1.pdf]

Supplementary Information for

Mass spectrometry imaging reveals new biological roles for choline esters and Tyrian purple precursors in muricid molluscs

David Rudd[1], Maurizio Ronci[2,3], Martin R. Johnston[4], Taryn Guinan[2], Nicolas H. Voelcker[2], Kirsten Benkendorff[5]*

[1]School of Biological Sciences, Flinders University, Bedford Park, SA 5042, Australia.

[2]Mawson Institute, University of South Australia, Mawson Lakes, SA 5095, Australia.

[3]Department of Clinical and Experimental Sciences, University G. D'Annunzio, Chieti-Pescara, Italy.

[4]Flinders Centre for Nanoscale Science and Technology, School of Chemical and Physical Sciences, Flinders University, Bedford Park, SA 5042, Australia.

[5]Marine Ecology Research Centre, Southern Cross University, P.O. Box 157, Lismore, NSW 2480, Australia

*Correspondence should be addressed to K. B. (Kirsten.Benkendorff@scu.edu.au)

Supplementary Results

Detection and structural elucidation of brominated indoles

The brominated indoles detected in *D. orbita* result from a series of enzymatic, oxidative and photolytic reactions (**Fig. 1**) from an ultimate precursor tyrindoxyl sulfate to produce the end product Tyrian purple (a mixture of 6'6-dibromoindigo and 6'6-dibromoindirubin)[1, 2, 3, 4, 5] The enzyme arylsulfatase is required to initiate the series of reactions by hydrolysing tyrindoxyl sulfate[2], which usually occurs outside the body. The structural features of the brominated indoles detected in this study are summarised in **Table S1**.

LC-MS

The main brominated indole extracted in the methanol fraction from the hypobranchial gland was the ultimate precursor compound tyrindoxyl sulfate[2] eluting at 6.59 min (major ions duplet peak *m/z* 336, 338; **Fig. S1** and **S2**) and tyrindoleninone[1] eluting at 10.96 min (major ion duplet peak *m/z* 256, 258; **Fig. S1** and **S2**). The remaining intermediate brominated indoles were extracted in the chloroform fraction from the hypobranchial gland including: tyriverdin[1] eluting at 11.67 min (major ion triplet peak centered *m/z* 419 and 465; **Fig. S1** and **S2**) and 6-bromoisatin[3] eluting at 6.35 min (major ion duplet peak *m/z* 224, 226; **Fig. S1** and **S2**). Tyrian purple 6'6-dibromoindigo [3, 6], the final brominated indole product, was detected in the chloroform fraction from the hypobranchial gland eluting at 14.34 min (major ion triplet peak *m/z* 419; **Fig. S1** and **S2**). Spectra for ESI-MS were run in negative mode causing a slight mass shift compared to DIOS-MS.

DIOS-MS imaging of brominated indoles

Tyrindoxyl sulfate, tyrindoleninone and 6'6-dibromoindigo (or the isomer 6'6-dibromoindirubin) were all detected in DIOS-MS based on the ion clusters for the mono and di-brominated structures from both semi-purified fractions (**Fig. S2**) and from tissue sections (**Fig. 1, 2, 3, S6**). Tyrian purple 6'6-dibromoindigo showed the most consistent pattern for localization in the hypobranchial gland and comparison of spectra showed a consistent triplet peak structure at *m/z* 419.88, 421.88 and 423.88 for Br^{79} Br^{79}, Br^{79} Br^{81} and Br^{81} Br^{81} (**Fig. 1**, **S2**). Tyrindoxyl hydrogen sulfate was detected consistently within the hypobranchial gland of the encapsulating female and post-reproductive female and two of the three pre-reproductive females (**Fig. S6**). Tyrindoleninone was detected in the pre-reproductive females localized in the medial hypobranchial gland of the encapsulating and post-reproductive

females (**Fig. S6)**. Ion intensity maps for the abundance of brominated indoles and murexine for all replicate samples are displayed in **Fig. S6**.

Egg capsule contents showed a slow chemical ripening process consistent with the enzymatic, oxidative and photolytic reaction seen in the medial hypobranchial gland (**Fig. 5** and **S7**), but extended over the 35 day intracapsular period, rather than the few hours required after liberation from the adult hypobranchial secretory tissue.

Detection and structural elucidation of murexine

LC-MS

The major compound eluting at 1.83 min provided a molecular ion $[M]^+$ peak in positive mode at 224.1 *m/z* (Table 1), consistent with the presence of murexine ($C_{11}H_{18}N_3O_2^+$; 224.284 MW). When subjected to a higher cone voltage to induce fragmentation, in ESI (35V), the eluting compound showed a fragment at 165 *m/z* which is the same loss of 59 that is observed in acetylcholine [7] (**Fig S3, S4**). Early intracapsular fluid contained murexine (**Fig. 5** and **Fig. S**7), which was absent in the late stage capsules.

NMR

Acetone extracts for murexine showed a combination of three compounds after removal of the bulk of crude contaminants[8] and column purification. The main components in NMR spectrum of the extracts vary with solvent, methanol dissolves less of the choline material. Shifts are also solvent dependant. Estimating amounts of the various components by integration in CD_3CN the charges balance (assuming NH that integrates for 2H is actually 1H). Shifts in correspondence to structure are summarised in **Table 2**.

Murexine:

^{1}H NMR (600 MHz, CD_3CN, 25 ˚C) δ 9.42 (2H, bs), 7.75 (1H, s), 7.61 (1H, d, J=15.7 Hz), 7.39 (1H, s), 9.49 (1H, d, J=15.7 Hz), 4.52-4.50 (2H, m), 3.61-3.60 (2H, m), 3.11 (9H, s).

Aromatic compound tyrindoxyl sulphate:

^{1}H NMR (600 MHz, CD_3CN, 25 ˚C) δ 7.66 (1H, d, J=15.7 Hz), 7.45 (1H, d, J=1.7 Hz), 7.13 (1H, dd, J=15.7, 1.7 Hz), 2.46 (3H, s).

Choline:

^{1}H NMR (600 MHz, CD_3CN, 25 ˚C) δ 3.90 – 3.88 (2H, m), 3.34-3.32 (2H, m), 3.06 (s, 9H).

Choline is often found with extracts of murexine as it is a hydrolysis product of the choline esters[9], can bind to tyrindoxyl sulphate and has been detected within ^{1}H NMR samples[10, 11]. The presence of tyrindoxyl sulphate would be likely as it is held as a salt of murexine, choline and other choline esters for storage prior to liberation from the medial hypobranchial gland[12].

References

1. Benkendorff, K., Bremner, J. B., Davis, A. R. Tyrian purple precursors in the egg masses of the Australian muricid, *Dicathais orbita*: A possible defensive role. *J Chem Ecol* **26**, 1037-1050 (2000).

2. Baker, J. T., Sutherland, M. D. Pigments of marine animals VIII. Precursors of 6,6'-Dibromoindigotin (Tyrian Purple) from the mollusc *Dicathais orbita* Gmelin. *Tetrahedron Lett*, 43 - 46 (1968).

3. Cooksey, C. J. Tyrian purple: 6,6 '-dibromoindigo and related compounds. *Molecules* **6**, 736-769 (2001).

4. Baker, J. T., Duke, C. C. Isolation from the hypobranchial glands of marine molluscs of 6-bromo-2,2-dimethylthioindolin-3-one and 6-bromo-2-methylthioindoleninone as alternative precursors to tyrian purple. *Tetrahedron Lett* **14**, 2481-2482 (1973).

5. Baker, J. T., Duke, C. C. Precursors of Tyrian Purple. In: *Food-Drugs from the Sea Proceedings* (eds. 1974).

6. Westley, C., Benkendorff, K. Sex-specific tyrian purple genesis: Precursor and pigment distribution in the reproductive system of the marine mollusc, *Dicathais orbita*. *J Chem Ecol* **34**, 44-56 (2008).

7. Keski-Rahkonen, P., Lehtonen, M., Ihalainen, J., Sarajarvi, T., Auriola, S. Quantitative determination of acetylcholine in microdialysis samples using liquid

chromatography/atmospheric pressure spray ionization mass spectrometry. *Rapid Commun Mass Spectrom* **21**, 2933-2943 (2007).

8. Roseghini, M., Severini, C., Erspamer, G. F., Erspamer, V. Choline esters and biogenic amines in the hypobranchial gland of 55 molluscan species of the neogastropod Muricoidea superfamily. *Toxicon* **34**, 33-55 (1996).

9. Bender, J. A. *et al.* Choline Esters in Marine Gastropods Nucella-Emarginata and Acanthina Spirata - New Choline Ester, Tentatively Identified as N-Methylmurexine. *Gen Pharmacol* **5**, 191-198 (1974).

10. Duke, C., Eichholzer, J., Macleod, J. The synthesis of the isomeric N-methyl derivatives of murexine. *Aust J Chem* **34**, 1739-1744 (1981).

11. Duke, C. C., Eichholzer, J. V., Macleod, J. K. N-Methylmurexine - naturally occuring marine compound. *Tetrahedron Lett*, 5047-5048 (1978).

12. Baker, J. T., Duke, C. C. Isolation of choline and choline ester salts of tyrindoxyl sulfate from marine mollusks *Dicathais orbita* and *Mancinella keineri*. *Tetrahedron Lett* **15**, 1233-1234 (1976).

Tables:

Supplementary Table 1. Summary of structural features for brominated indoles and choline esters detected in *Dicathais orbita* hypobranchial tissue from LC-MS, DIOS-MS and ^{1}H NMR (tyrindoxyl sulfate and murexine from this study, intermediate brominated indoles from Benkendorff et al. 20001).

Supplementary Table 2. Summary of ^{1}H-NMR and ^{13}C-NMR fingerprinting from reproductive female acetone extract

Figures:

Supplementary Figure 1. Liquid chromatogram of brominated indoles detected at 300 and 600 nm.

Supplementary Figure 2. MS-MS spectra for brominated indoles detected from hypobranchial gland extracts of *Dicathais orbita* by ESI LC-MS (in negative mode) and representative DIOS-MS.

Supplementary Figure 3. LC-MS of the capsule gland extract from a reproductively active female *Dicathais orbita*, containing (a) murexine.

Supplementary Figure 4. Murexine structural fragmentation seen in DIOS mass spectrum from reproductively active *Dicathais orbita* female capsule gland.

Supplementary Figure 5. Region specific ion intensities for murexine *m/z* 224.16.

Supplementary Figure 6. DIOS-MSI of reproductively active female *Dicathais orbita.*

Supplementary Figure 7. *Dicathais orbita* reproductive adults and early stage egg capsules with DIOS-MS of capsule contents extract (Photo by Rudd, D.).

Video:

Supplementary Video 1. *Dicathais orbita* larval motility bioassay using 50mg/L purified murexine. Murexine was introduced after a seawater addition to assess response to external stimuli. Larvae motility was recorded using 30 sec videos at 0, 5, 10, 20, 30 and 60 min intervals (online: https://youtu.be/rlCvyyhnXAE) .

Supplementary Table 1. Summary of structural features for brominated indoles and choline esters detected in *Dicathais orbita* hypobranchial tissue from LC-MS, DIOS-MS and ^{1}H NMR (tyrindoxyl sulfate and murexine from this study, intermediate brominated indoles from Benkendorff et al. 2000[1])

Structure	Compound	Formula	MW	RT [min]	Major ions m/z		^{1}H NMR
					LC-MS	DIOS-MS	
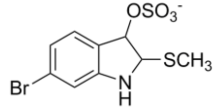	tyrindoxyl sulfate	$C_9H_7BrNO_4S_2^-$	337.196	6.59	336, 338	339.98, 340.99, 341.98, 342.98	δH 9.76s (1H), 7.14m (3H), 2.42s (3H)
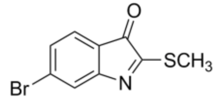	tyrindoleninone	C_9H_6BrNOS	256.124	10.96	256, 258	255.95, 256.94, 257.94, 258.95	(CD_3CN), δH 7.46 (1H, dd, *J*=0.5, 1.4 Hz), 7.42 (1H, dd, *J*=0.5, 7.6 Hz), 7.39 (1H, dd, *J*=7.6, 1.4 Hz), 2.63 (3H, s)
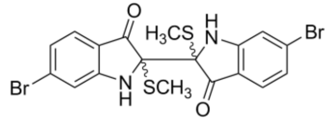	tyriverdin	$C_{18}H_{14}Br_2N_2O_2$	514.264	11.67	417, 419, 421, 463, 465, 467, 511, 513, 515	n.d.	(DMSO-d_6), δH 7.46d (8.4), 7.27d (1.5), 6.96dd (8.4, 1.5), 1.90s
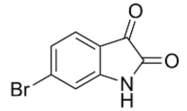	6-bromoisatin	C_8H_4BrNOS	226.029	6.35	224, 226	n.d.	(CD_3CN), δH 8.96 (1H, s), 7.44 (1H, d, *J*=8.08 Hz), 7.30 (1H, dd, *J*=1.64, 8Hz), 7.19 (1H, d, *J*=1.6 Hz)
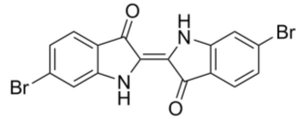	6,6'-dibromoindigo	$C_{16}H_8Br_2N_2O_2$	420.06	14.34	417, 419, 421	418.88, 419.88, 420.88, 421.88, 422.88, 423.88, 424.88	(N;N'-bistrifluoroacetyl derivative, $CDCl_3$), δH 8.3d (1.2), 8.06d (8.4), 7.95d (7.7), 7.79td (7.4, 1.3), 7.75d (8.1), 7.56dd (8.1, 1.4), 7.42t (7.4)
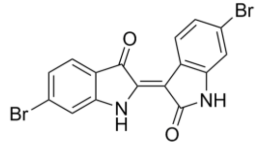	6,6'-dibromoindirubin	$C_{16}H_8Br_2N_2O_2$	420.06	n.d.	417, 419, 421	418.88, 419.88, 420.88, 421.88, 422.88, 423.88, 424.88	(DMSO-d_6), δH 11.2brs, 11.1brs, 8.67d (8.5), 7.68d (1.6), 7.62d (8.1), 7.22dd (8.5, 1.7), 7.20dd (7.9, 1.6), 7.05d (1.8)
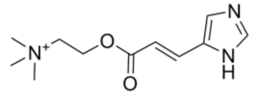	murexine	$C_{11}H_{18}N_3O_2^+$	224.284	1.83	224.1	224.17, 225.17	(D_2O, CD_3COOD), δH 7.05 (1H, d, *J* =16), 8.08 (1H, d, *J* =16), 8.28 (1H, s), 9.28 (1H, s), 5.10 (2H, m), 4.23 (2H, m), 3.77 (9H, s)

Table 2 ^{1}H-NMR assignment for purified acetone extract containing murexine, choline and counterion tyrindoxyl sulfate. [a]Relative area is influenced by compound integration for counter ion tyrindoxyl sulfate, where in CD_3CN the charges balance.

compound	δ (p.p.m.)	mutiplicity		relative area	Assignment
**a - Murexine**	3.11	s		9	1
	3.60 - 3.61	m		2	2
	4.50 - 4.52	m		2	3
	6.49	d	J = 15.7 Hz	1	4
	7.61	d	J = 15.7 Hz	1	5
	7.39	s		1	6
	7.75	s		1	7
	9.42	bs		2[a]	8
**b - Choline**	3.06	s		9	1
	3.32 - 3.34	m		2	2
	3.88 - 3.90	m		2	3
**c - Tyrindoxyl sulfate**	2.46	s		3	1
	7.13	dd	J = 15.7 Hz, 1.7 Hz	1	2
	7.45	d	J = 1.7 Hz	1	3
	7.66	d	J = 15.7 Hz	1	4

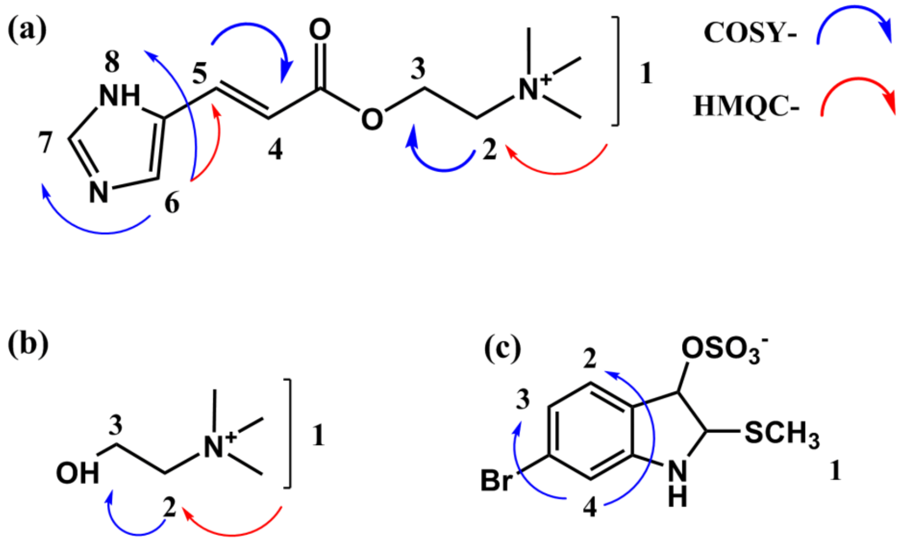

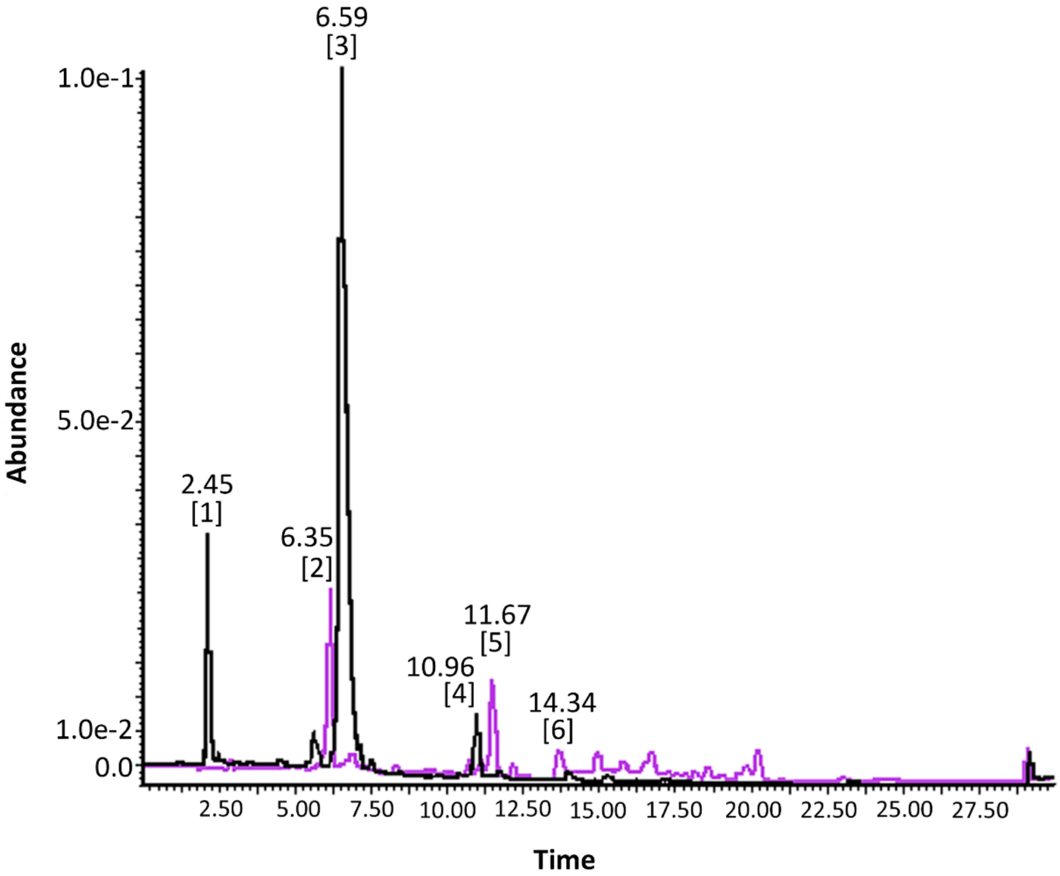

Supplementary Figure 1. Liquid chromatogram of brominated indoles detected at 300 and 600 nm. (Black line) methanol fraction containing [1] murexine eluting at 2.45 min, [3] tyrindoxyl sulphate eluting at 6.59 min and [4] tyrindoleninone eluting at 10.96 min. (Purple line) chloroform fraction containing [2] 6-bromoisatin eluting at 6.35 min, [5] tyriverdin eluting at 11.67 min and [6] 6'6-dibromoindigo (Tyrian purple) eluting at 14.34 min.

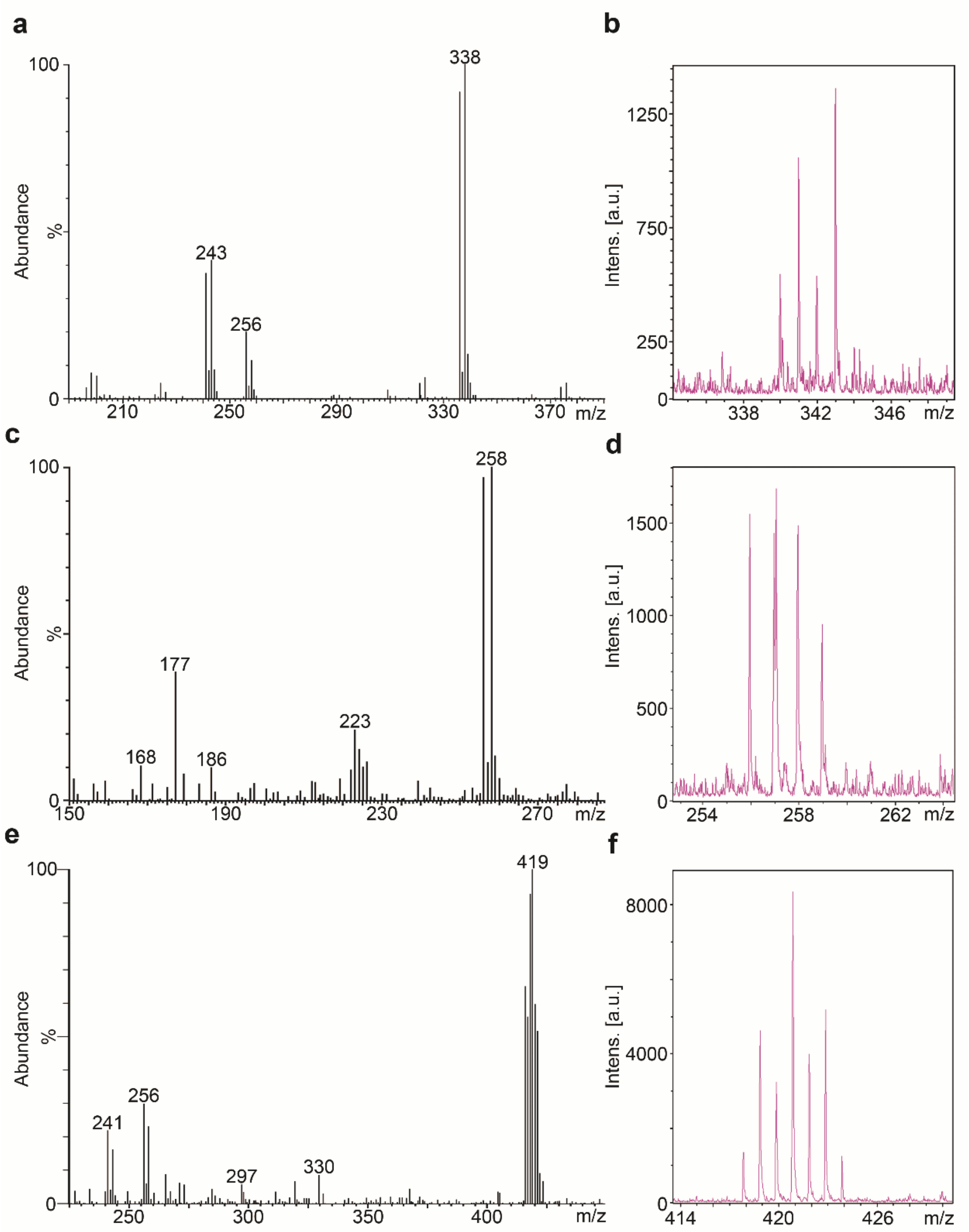

Supplementary Figure 2. MS-MS spectra for brominated indoles detected from hypobranchial gland extracts of Dicathais orbita by ESI LC-MS (in negative mode) and representative DIOS-MS (see Supplementary Figure 1 and Table 1; in positive mode); (a) MS-MS spectrum for precursor compound tyrindoxyl sulfate, (b) corresponding DIOS mass spectrum of tyrindoxyl hydrogen sulfate [*m/z* 339.98, 340.99, 341.98, 342.98 ± 0.05 [M+H]+], (c) MS-MS spectrum for intermediate tyrindoleninone, (d) corresponding DIOS mass spectrum of tyrindoleninone [*m/z* 255.95, 256.94, 257.94, 258.95 ± 0.05 [M]+], (e) MS-MS spectrum for Tyrian purple 6,6'-dibromoindigo, (f) corresponding spectrum in DIOS-MS of 6,6'-dibromoindigo [*m/z* 419.88, 421.88, 423.88, ± 0.05 [M+2H]+].

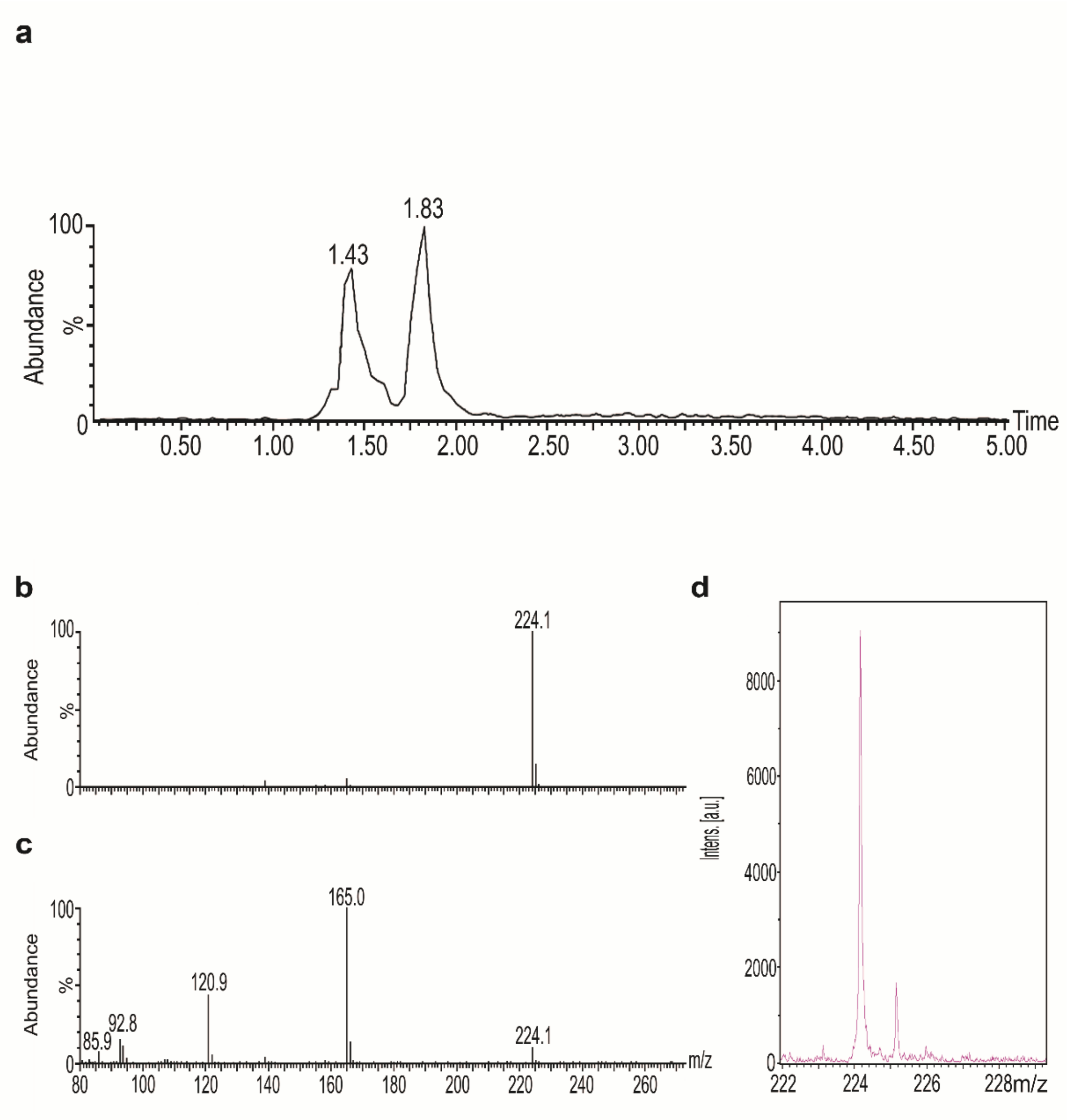

Supplementary Figure 3. LC-MS of the capsule gland extract from a reproductively active female *Dicathais orbita*, containing (**a**) murexine eluting at 1.83 min in the liquid chromatograph; (**b**) MS-MS spectra of murexine; (**c**) collision induced dissociation of murexine with *m/z* 165 representing $N^{+}(CH_3)3$ loss of 59 and (**d**) representative DIOS mass spectra of murexine [*m/z* 224.17, 225.17 ± 0.02 $[M+H]^{+}$]

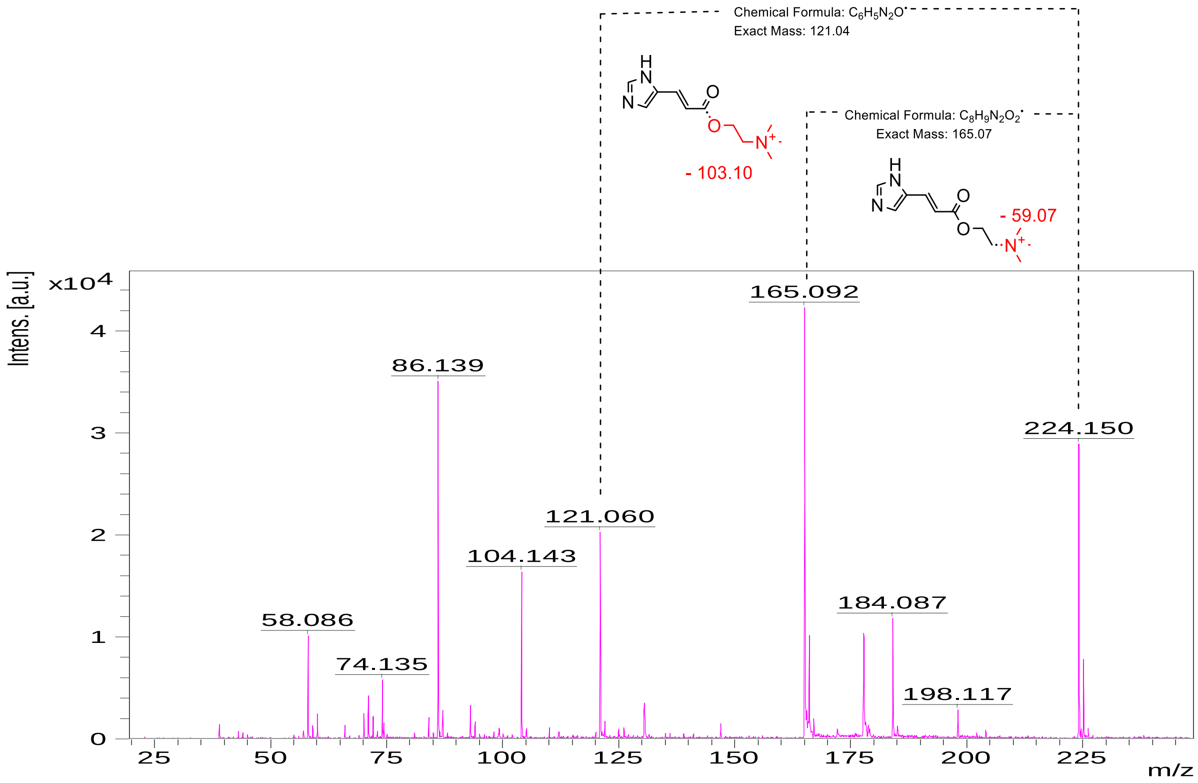

Supplementary Figure 4. Murexine structural fragmentation seen in DIOS mass spectrum from reproductively active *Dicathais orbita* female capsule gland. *m/z* 224.15 represents the major ion detected in both DIOS-MS and LC-MS (**Supplementary Fig. 4**).

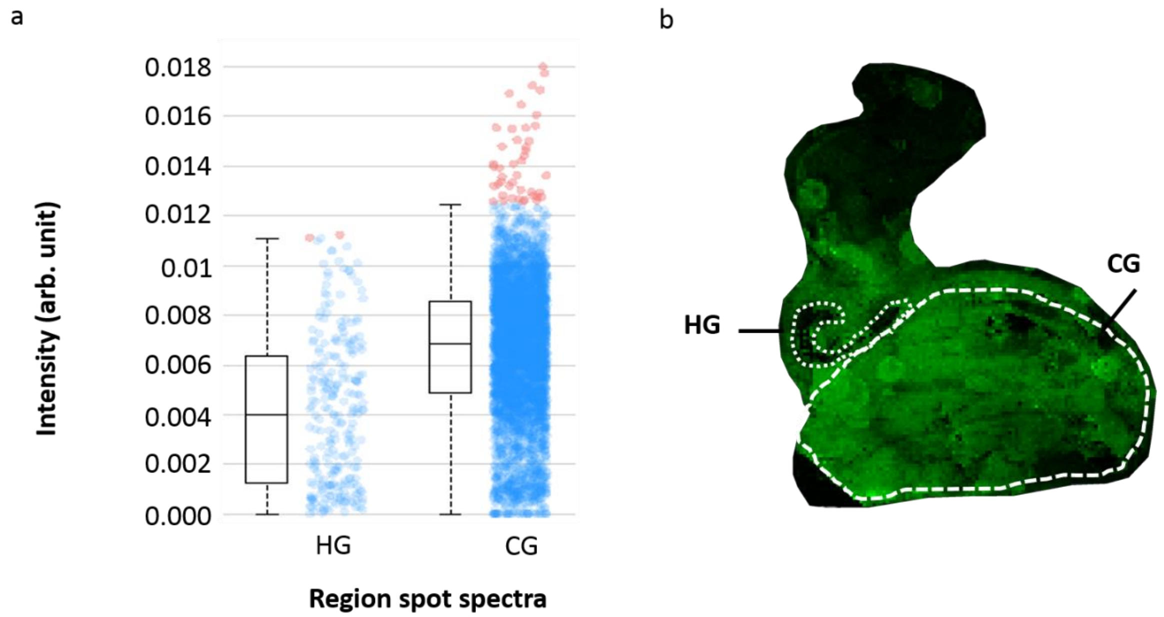

Supplementary Figure 5. Region specific ion intensities for murexine *m/z* 224.16, where a) each dot represents a spot in the tissue section plotted against intensity value for the hypobranchial gland (HG) and the capsule gland (CG) with quartile distribution shown with associated box plots; blue dots represent intensity spots that lie within the normal distribution and reds spots represent outlier intensity spots. The corresponding female *m/z* 224.16 distribution detected in DIOS-MSI is shown in b) with specific regions indicated.

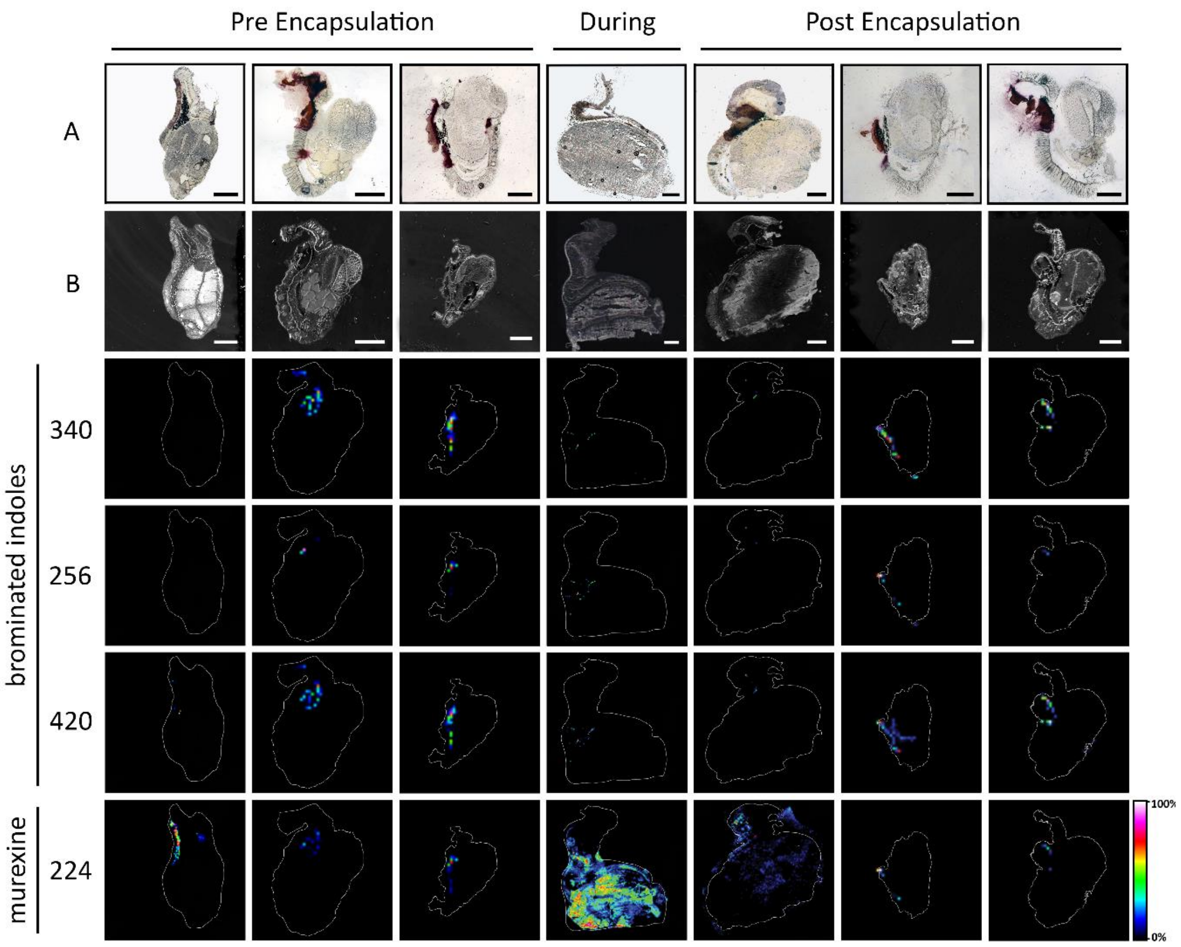

Supplementary Figure 6. DIOS-MSI of reproductively active female *Dicathais orbita*. 15µm thick cryo-sections of the central hypobranchial gland with attached capsule gland were imprinted onto functionalised porous silicon chips and mass spectrometry imaged. Pre Encapsulation) female sections sampled 30 days prior to the start of the standard breeding season. During) a female section sampled during egg encapsulation. Post Encapsulation) female sections sampled 14 days post egg encapsulation. A) serial histological sections, where (hg) indicates the medial hypobranchial gland and (cg) indicates the capsule gland. B) scanned imaged of gland tissue cryo-section on pSi chip. *m/z* 340 is tyrindoxyl hydrogen sulphate $[M+H]^+$. *m/z* 256 is tyrindoleninone $[M+H]^+$. *m/z* 421 is Tyrian purple (6'6-dibromoindigo) $[M+H]^+$. *m/z* 224 is murexine $[M]^+$. Scale bar set to 2 mm. Intensities of ions in the imaged sections are colour coded using a heat map.

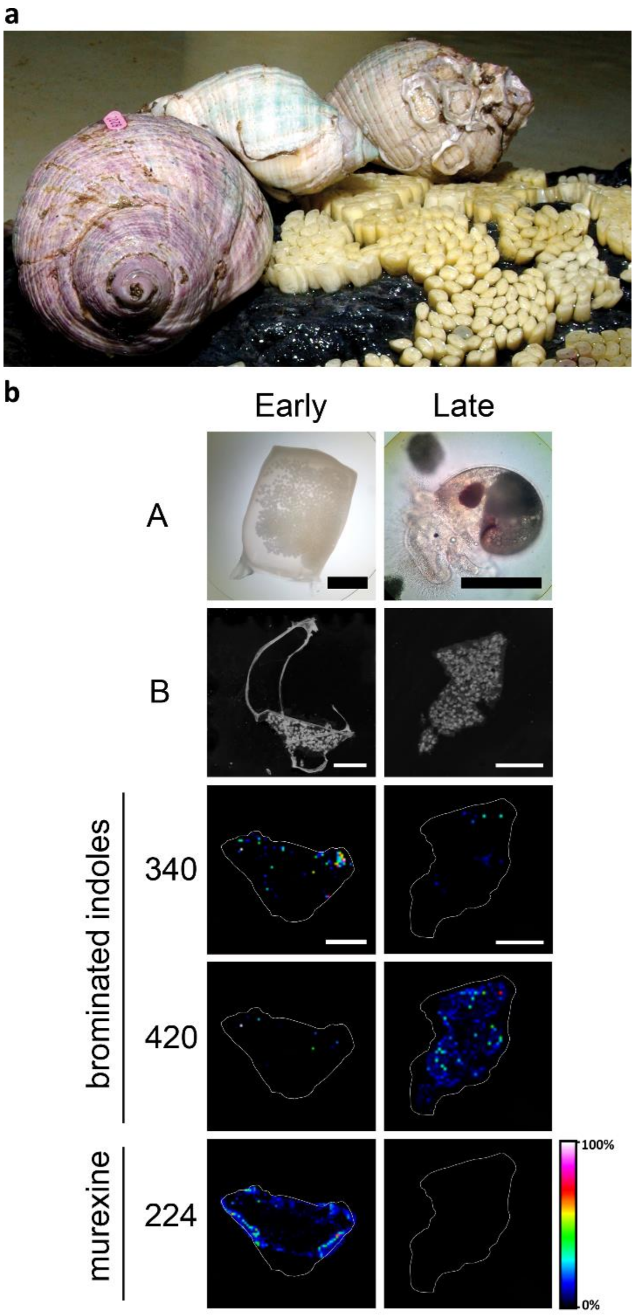

Supplementary Figure 7. *Dicathais orbita* reproductive adults and early stage egg capsules with DIOS-MS of capsule contents. (a) Reproductive adults during the encapsulation of larvae and early stage capsules adhered to substrate (Photo by Rudd, D.). (b) DIOS-MSI of an early stage capsule sampled immediately post deposition and late stage capsule, 35 days post deposition. A is a microphotograph of the entire capsule and late stage larvae. B is a scanned imaged of the cryo-section capsules imprinted onto pSi chip. *m/z* 340 is tyrindoxyl hydrogen sulphate $[M+H]^+$. *m/z* 420 is Tyrian purple (6’6-dibromoindigo) $[M+H]^+$. *m/z* 224 is murexine $[M]^+$. Scale bar set to 1mm. Intensity of ions detected in egg capsule contents are colour coded using a heat map.
